# FLRT3 Marks Direction-Selective Retinal Ganglion Cells That Project to the Medial Terminal Nucleus

**DOI:** 10.3389/fnmol.2021.790466

**Published:** 2021-12-09

**Authors:** Tobias Ruff, Christian Peters, Akihiro Matsumoto, Stephan J. Ihle, Pilar Alcalá Morales, Louise Gaitanos, Keisuke Yonehara, Daniel del Toro, Rüdiger Klein

**Affiliations:** ^1^Department of Molecules, Signaling, and Development, Max Planck Institute of Neurobiology, Martinsried, Germany; ^2^Laboratory of Biosensors and Bioelectronics, ETH Zürich, Zurich, Switzerland; ^3^Department of Biomedicine, Nordic-EMBL Partnership for Molecular Medicine, Danish Research Institute of Translational Neuroscience – DANDRITE, Aarhus University, Aarhus, Denmark; ^4^Department of Biological Sciences, Faculty of Medicine, Institute of Neurosciences, IDIBAPS, CIBERNED, University of Barcelona, Barcelona, Spain

**Keywords:** FLRT3, RGC, MTN, ON direction selective, downward movement

## Abstract

The mammalian retina extracts a multitude of diverse features from the visual scene such as color, contrast, and direction of motion. These features are transmitted separately to the brain by more than 40 different retinal ganglion cell (RGC) subtypes. However, so far only a few genetic markers exist to fully characterize the different RGC subtypes. Here, we present a novel genetic Flrt3-CreERT2 knock-in mouse that labels a small subpopulation of RGCs. Using single-cell injection of fluorescent dyes in Flrt3 positive RGCs, we distinguished four morphological RGC subtypes. Anterograde tracings using a fluorescent Cre-dependent Adeno-associated virus (AAV) revealed that a subgroup of Flrt3 positive RGCs specifically project to the medial terminal nucleus (MTN), which is part of the accessory optic system (AOS) and is essential in driving reflex eye movements for retinal image stabilization. Functional characterization using *ex vivo* patch-clamp recordings showed that the MTN-projecting Flrt3 RGCs preferentially respond to downward motion in an ON-fashion. These neurons distribute in a regular pattern and most of them are bistratified at the level of the ON and OFF bands of cholinergic starburst amacrine cells where they express the known ON-OFF direction-selective RGC marker CART. Together, our results indicate that MTN-projecting Flrt3 RGCs represent a new functionally homogeneous AOS projecting direction-selective RGC subpopulation.

## Introduction

Retinal ganglion cells (RGCs) transmit visual information captured by the eyes to different regions in the brain. Distinct RGC subtypes respond with different activity patterns to the same set of visual stimuli (Baden et al., 2016). The feature selectivity of each RGC subtype is established by the various patterns of connections with different types of amacrine and bipolar cells in the inner plexiform layer (IPL). One such feature is the selective activity in response to motion stimuli into a certain direction (Vaney et al., 2012). The detection of motion direction in the visual system is essential to drive the optokinetic reflex to stabilize the retinal image in response to the slow head or eye movements (Yoshida et al., 2001; Yonehara et al., 2016). In the brain, these stabilization signals are processed by different nuclei of the accessory optic system (AOS): the medial and lateral terminal nuclei (MTN and LTN), and the optic tract and the dorsal terminal nucleus (NOT/DTN) (Yonehara et al., 2009). It has been shown that three types of ON and one type of ON-OFF direction-selective RGC that project to the AOS are tuned to either upward (ON), downward (ON), or forward (ON, ON-OFF) direction (Dhande et al., 2013). To fully understand how each channel contributes to the smooth adaptive eye movements of the optokinetic reflex, it is essential to gain specific genetic access to each of the four input channels. Generally, all four types of AOS projecting RGCs are genetically accessible using the Hoxd10 mouse line (Dhande et al., 2013). So far only the SPIG1 and the Pcdh9-Cre line were shown to each specifically label one type, the dorsal (ON), or ventral (ON) selective RGCs (Yonehara et al., 2009; Lilley et al., 2019). However, we still lack specific genetic access to each of the remaining two AOS projecting RGCs.

Previous work from our lab using the fibronectin leucine rich-repeat transmembrane (Flrt) protein, showed that these cell adhesion molecules lead to cortex folding and altered distribution of pyramidal neurons during cortical development (Seiradake et al., 2014) in the mouse (del Toro et al., 2017). We observed that Flrt3 is expressed in early postnatal stages (Seiradake et al., 2014), and in the adult retina (Visser et al., 2015), and thought it could thus be interesting to evaluate it as a genetic marker. Here, using a novel, inducible CreERT2 knock-in line inserted into the *Flrt3* locus on chromosome 2 and a combination of morphological, molecular, and electrophysiological characterizations, we identified four morphologically distinct RGC types. Anterograde tracings revealed that one Flrt3-positive RGC subtype specifically projects to the medial terminal nucleus (MTN) of the AOS. Functional characterizations of the MTN-projecting Flrt3-subtype revealed that they are ON-RGCs and respond preferentially to downward moving stimuli. Interestingly, most MTN-projecting Flrt3-RGCs are bistratified in the ON and OFF-ChAT band, which has not been described before. Thus, our results indicate the presence of an additional fifth AOS projecting direction-selective RGC subtype.

## Materials and Methods

### Mouse Lines

The following mouse lines used in this work have been previously described and were back-crossed to C57BL/6J mice: FLRT1^LacZ^ (EUCOMM), Flrt2^LacZ^ (EUCOMM), Flrt3^LacZ^ (Egea et al., 2008), Ai9^lsl–tdTomato^ [B6.Cg-Gt(ROSA)26Sor^TM9(CAG–tdTomato)Hze^/J] (Madisen et al., 2010).

Briefly, the Flrt3^Cre/ERT2^ knock-in line used in this study was generated by homologous recombination in embryonic stem (ES) cells using a replacement-type targeting vector. The targeting construct for Flrt3 was generated by inserting a sequence containing part of the 5′UTR of the exon III, the CreERT2 sequence, an SV40 polyadenylation signal and a loxP-flanked neo cassette into a vector containing 3.8 kb and 6 kb homology arms surrounding exon III of the Flrt3 gene and the thymidine kinase (TK) cassette. The sequence flanking the insertion site in the 5′UTR is 5′ TAACAGAAGCTACCTGCTATAAT 3′. The sequence flanking the insertion site in the 3′UTR is 5′ TGAGAGAAGCAATGTACTGTACATTT 3′.

Embryonic stem cell cultures, electroporation, and selection were carried out according to standard protocols. Screening and homologous recombination on both arms of the constructs was assessed by Southern blot in the targeted ES cells. Germline transmission was achieved with at least two independent ES cell clones. Mice were maintained in a C57BL/6J background. They will be deposited in the Mutant Mouse Resource and Research Centers (MMRRC) for distribution. Animals were kept and used according to the regulations of the Regierung von Oberbayern.

The FLRT3-CreERT2 line was crossed to the R26-tdTomato (Ai9) line [B6.Cg-Gt(ROSA)26Sortm9(CAG-tdTomato)Hze/J] (Madisen et al., 2010).

### 4-Hydroxytamoxifen Administration

4-hydroxytamoxifen (Sigma, ref: H-7904) solution was prepared according to the manufacturer’s protocol (Chevalier et al., 2014). To induce Cre expression in the Flrt3-CreERT2 line, adult mice (>P60) of both sexes were injected intraperitoneally with 60 μg/g 4-Hydroxytamoxifen.

### Retina Histology

For retina whole-mount preparation, eyes were first enucleated and a small hole was made in the dorsal side to maintain correct orientation after dissection. Next, the cornea and lens were removed by cutting along the ora serrata and the retina cleaned of any residues from the vitreous. Finally, the retina was detached from the eyecup and transferred onto a cell culture insert (Millipore, Cat. no. PICMORG50). To allow a flat mount of the retina, 4 incisions were made. The deepest incision marked the dorsal side. Retinas were fixed on the membrane for at least 30 min in 4% paraformaldehyde (PFA).

### Brain Histology

For analysis of retinofugal projections, the animals were deeply anesthetized with an overdose of Ketamine (medistar)/Xylazine (Bernburg) (1.6%/0.08%). Mice were transcardially perfused for 10 min at a speed of 1 ml/min with ice-cold PBS followed by 10 min of ice-cold 4% PFA (10% stock solution, Electron Microscopy Sciences, 15712) in PBS. Brains were post-fixed in 4% PFA for 24–48 h and subsequently stored in PBS (0.02% Sodium azide). Brains were cut at 100 μm slices using a vibratome (Leica). Retinas were dissected after transcardial perfusion.

### Immunohistochemistry

For immunostainings, retinas were fixed in 4% PFA and transferred to a permeabilizing and blocking solution containing 0.3% Triton X-100, 5% donkey serum, 0.2% bovine serum albumin, 0.2% lysine, and 0.2% glycine for at least 24 h. After three washes in PBS, retinas were incubated with one of the following primary antibodies (all antibodies were used at a dilution of 1:100, unless otherwise stated): guinea pig anti-RBPMS (ABN1376, Millipore, United States); rabbit anti-Calbindin-D-28k 1:200 (702411, Thermo Fisher Scientific, United States); rabbit anti-CART 1:300 (14547S, Cell Signaling Technology, United States); rabbit anti-ChAT (AB-N34AP, ATS, United States); rabbit anti-Parvalbumin (PV27, Swant, Switzerland); rabbit anti-Satb1 (ab70004 abcam); mouse anti-SMI32 (801701, BioLegend, United States); mouse anti-Brn3c (sc-81980, Santa Cruz Biotechnology, United States). The tissue was incubated for 1–3 days with the primary antibodies (in 0.3% Triton X-100, 2% bovine serum albumin, 0.02% sodium azide in PBS). After 3 washes in PBS, the secondary antibody (in 0.3% Triton X-100, 3% donkey serum in PBS) was added for at least 1 day.

### X-Gal Staining

For X-Gal staining, the retina was dissected from the eye and fixed for 30 min in an X-Gal fixative solution (0.2% Glutaraldehyde, 1% PFA in PBS, 5 mM EGTA, and 2 mM MgCl_2_ and 0.02% NP40). Brains were fixed for 1–3 h. Retinas and brain regions were stained for beta-galactosidase activity by incubating them for 5–10 h in a 1 mg/ml X-gal solution containing 5 mM K_4_Fe(CN)_6_ and 5 mM K3Fe(CN)6. Retinas were postfixed for 10 min in 4% PFA.

### Single Retinal Ganglion Cell Injections

For single-cell reconstructions, retinas of FLRT3-CreERT2 × R26-tdTomato (Ai9) mice were dissected from the eye and mildly fixed in 4% PFA for 15 min. FLRT3+ neurons were identified by Tomato expression and injected with 4% Lucifer yellow dissolved in ACSF using a sharp electrode. Sharp electrodes were pulled from borosilicate glass capillaries to a final resistance of 200 Ω (Science Products GP150F-8P) and mounted on a motorized patch-clamp manipulator. The dye was expelled using a positive current of 1–2 nA at 100 ms pulses for about 2 min (90% duty cycle).

### Intravitreal Virus Injections

Mice were anesthetized intraperitoneally with a mixture of fentanyl [0.05 mg/kg, SIGMA-ALDRICH Chemie GmbH (800021)], midazolam [5 mg/kg, SIGMA-ALDRICH Chemie GmbH (800021)], and medetomidine [5 mg/kg, TCI Deutschland GmbH (800142)]. On the day of surgery, and on two subsequent days, carprofen [5 mg/kg, Zoetis (Rimadyl)] was also administered as an additional analgesic. Surgical instruments were heat-sterilized and washed with ethanol. The 7000 series Neuros Hamilton syringe with a 32G blunt needle was rinsed several times with distilled H_2_O, then ethanol, and again with distilled H_2_O. The animal was then fixated in a stereotaxic setup (Kopf instruments) and the eye that was injected second was kept damp with eye drops (Oculotect, Novartis). Briefly, the surgery was performed by cautiously protruding the eye from the eye socket and punctured at the ora serrata using a 30-gauge needle. Next, 1 μl of the adeno-associated virus (AAV) (rAAV2/CAG-GFP, rAAV2/Flex-GFP, rAAV2/Flex-tdTomato) was injected using a Hamilton syringe controlled by a micromanipulator (M3301R, WPI) and inserted into this opening at an oblique angle to avoid damaging the lens. After injection, the syringe was left in place for 4 min to allow the virus to disperse. After removing the Hamilton syringe, the eye was covered with eye cream (Isopto-Max, Novartis) and the procedure was repeated with the other eye. After 3 weeks, 2 μl of Cholera Toxin Subunit B conjugated with Alexa Fluor 647 (CTB647, C34778, Invitrogen, United States) (1%) was injected using a similar procedure. For histological analysis the animals were sacrificed as described previously in “brain histology” and perfused using 4% PFA 24 h later. The brain and retina were postfixed in 4% PFA for 24 and 1 h, respectively.

### Stereotaxic Surgeries

Mice were anesthetized using isoflurane (Cp-pharma, 1.5–2%) and placed in a stereotaxic setup (Kopf Instruments). Body temperature was maintained using a heating pad at 37°C, and an analgesic was injected subcutaneously (5 mg/kg, Rimadyl, Zoetis). Viruses (pAAV-FLEX-tdTomato (Addgene), rAAV5-hsyn-EYFP (UNC GTC vector core, United States)), or CTB (cholera toxin subunit B, Invitrogen) were injected into the MTN (AP −2.65, ML ± 0.95, DV −5) using glass pipettes (#708707, BLAUBRAND intraMARK).

### Electrophysiology

Mice were dark-adapted overnight and sacrificed by cervical dislocation. The retinas were dissected immediately after and transferred to oxygenated (95% O_2_ and 5% CO_2_) Ames solution at room temperature (∼22°C). The retina was placed onto an Anodisc filter membrane (Whatman, WHA68096022) with the ganglion cell layer facing upward and transferred into the recording chamber. The ventral part of the retina was marked for orientation. Flrt3-tomato neurons were visualized under brief fluorescent illumination using a microscope equipped with IR–DIC optics (Olympus BX51). All electrophysiological recordings were performed with constant superfusion of carbogenated Ames solution at 30–32°C. Whole-cell-current-clamp or cell-attached recordings were performed with a MultiClamp 700B amplifier and a Digidata 1550 digitizer (Molecular Devices). For current-clamp recordings, a patch pipette with a resistance of 4–6 MΩ was filled with an intracellular recording solution (130 mM potassium gluconate, 10 mM KCl, 2 mM MgCl_2_, 10 mM HEPES, 2 mM Na-ATP, 0.2 mM Na_2_GTP, 0.2% neurobiotin, pH7.35, and 290 mOsm). For cell-attached recordings, the patch pipette was filled with Ames solution.

Blue light stimuli were presented using a DLP projector (DLP^®^ LightCrafter 4500) through a 4× objective. Direction selectivity was analyzed by presenting a moving grid (bar width = 285 μm, speed = 570 μm/s, time = 2 s) in eight different directions. Only a few neurons were unresponsive to visual stimuli and thus discarded. Responses to stationary centered flashing dots of increasing size [(50–1,200 μm), time = 2 s] were recorded.

For firing adaptation calculation, we used the formula [1 – (T_last_/T_initial_)], where T_last_ is the time interval between the last two action potentials and T_initial_ is the time interval between the first two action potentials. Consequently, a value of 0 represents no change in spike frequency, while higher values represent a decay in the spiking rate of a neuron.

### Cell Distribution Analysis

For cell distribution analysis, cells of interest were manually marked using the cell counter plugin from Fiji. To evaluate the cell distribution regularity, density recovery profiles (DRP) were calculated using a custom made python script. To calculate the density recovery profile, the amount of cells (N) within different radii (r) of each cell was counted and divided by the area (A) covered between radius r and r + ∂r. The DRP was then calculated as density p(r) = N(r + ∂r)/(A(r + ∂r). We defined the regularity index as the mean nearest neighbor distance divided by the standard error of the mean.

### Cell Morphology Analysis and Classification

For cell morphology analysis, each cell was traced in a semi-automatic fashion using the Fiji plugin Simple Neurite tracer. To extract morphology features (such as stratification depth, number of branching points, and total neurite length) a custom-made python script was used based on the NeuroM toolkit from the Blue Brain Project^1^.

### Inner Plexiform Straightening to Enable Stratification Analysis

For stratification analysis, z-stacks of retinal sections (vibratome cut cross sections orthogonal to the retinal cell layers) were imaged using a Leica SP8 microscope. A custom-made python script was used to straighten the curved retina sections. The script used the DAPI staining of the ganglion cell layer and inner nuclear layer to define the boundaries of the inner plexiform layer. Afterward, the sections were straightened using the bilinearly blended Coons patch using the previously established boundaries of the inner plexiform layer (Farin and Hansford, 1999). Retinal sections in which straightening failed were discarded from the analysis.

### Data Analysis

Data and statistical analyses were performed using GraphPad Prism v5, Python 3.6, and Clampfit (Molecular Devices, United States). Significance levels were analyzed using a two-tailed unpaired Student’s *t*-test when comparing two groups (Figure 3C), or one-way ANOVA test with Tukey’s *post hoc* test when comparing multiple groups (Figures 5D,E), where *P*-values represent *p < 0.05; ^**^p < 0.01; ^***^p < 0.001. All data are represented as the mean ± SEM. All sample sizes and definitions are provided in the figure legends. The python scripts used in this study are available upon request.

**FIGURE 1:**
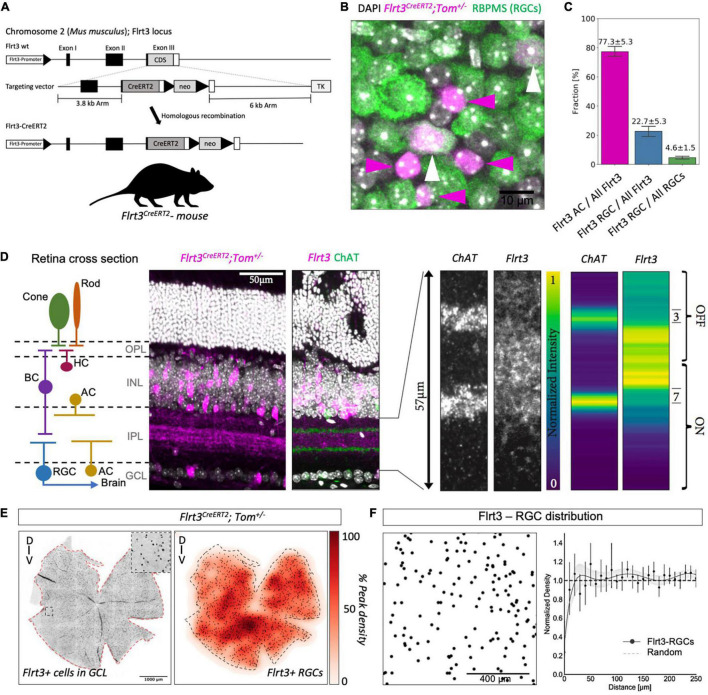
Flrt3 neuronal distribution in the mouse retina. **(A)** Scheme of the Flrt3-CreERT2 mouse. **(B)** Immunostaining showing Flrt3+ cells in magenta (tomato), RGCs in green (RBPMS), and the nucleus in white (DAPI). The white arrows indicate FLRT3-RGCs and the magenta arrows FLRT3-amacrine cells. **(C)** Plot showing the fractions of Flrt3-amacrine (AC) and FLRT3-RGCs in the Flrt3-CreERT2 mouse. Two complete retinas were counted and another three representative regions of interest (ROIs) of another two retinas were included in the quantification. **(D)** Retina sections of *Flrt3-CreERT2;Tom^±^* mouse showing Flrt3 cells in purple and ChAT bands in green. The left panel shows the anatomical structure and cell types of a typical retinal section. The right panel shows a zoom of the IPL region and normalized intensity of ChAT and Flrt3 signals. AC, amacrine cells; BC, bipolar cells; HC, horizontal cells; RGC, retinal ganglion cell; OPL, outer plexiform layer; INL, inner nuclear layer; IPL, inner plexiform layer; GCL, ganglion cell layer. **(E)** Representative retina density profile (%) of *Flrt3-CreERT2;Tom*^±^ mice. The left panel shows the distribution of Flrt3+ cells in the ganglion cell layer. The right panel shows the density of Flrt3+ RGCs identified from the left panel. *N* = 2 retinas from 2 mice **(F)** Random distribution of Flrt3-RGCs. Left panel: Flrt3-RGC distribution in a ROI of a retina region. Each dot represents a Flrt3+ RGC. Right panel: Density recovery profile of Flrt3+ RGC showing a random distribution. Graph shows Mean value ± SEM. Line represents a 7th order fit of the mean values. Shaded area is the 99% confidence interval. *N* = 4 retinas from 2 mice.

**FIGURE 2:**
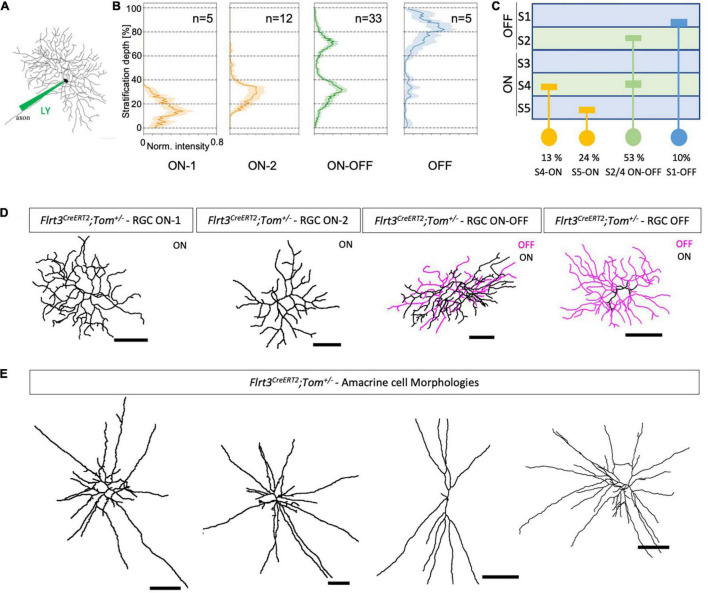
Dye-filled RGCs revealing their morphology and stratification. **(A)** Injection of a fluorescent dye [Alexa 488 or Lucifer yellow (LY)] in Flrt3-RGCs with a borosilicate glass pipette using a patch-clamp setup and filling the whole neuron to assess the morphology of the cells. **(B)** Dendritic stratification pattern of Flrt3-RGCs in the IPL for ON, ON-OFF, and OFF cells. Stratification depth between the inner limiting borders of the GCL (=0%) and INL (=100%) defined by DAPI staining. *N* = 5 retinas from 4 mice. **(C)** Scheme summarizing the stratification pattern and their percentages. **(D)** Representative morphologies of Flrt3 RGCs stratifying in different IPL layers that were filled with Lucifer yellow and reconstructed using the Fiji Simple Neurite Tracer plugin. **(E)** Different morphologies of Flrt3 amacrine cells of the INL. Scale bars = 50 μm.

**FIGURE 3:**
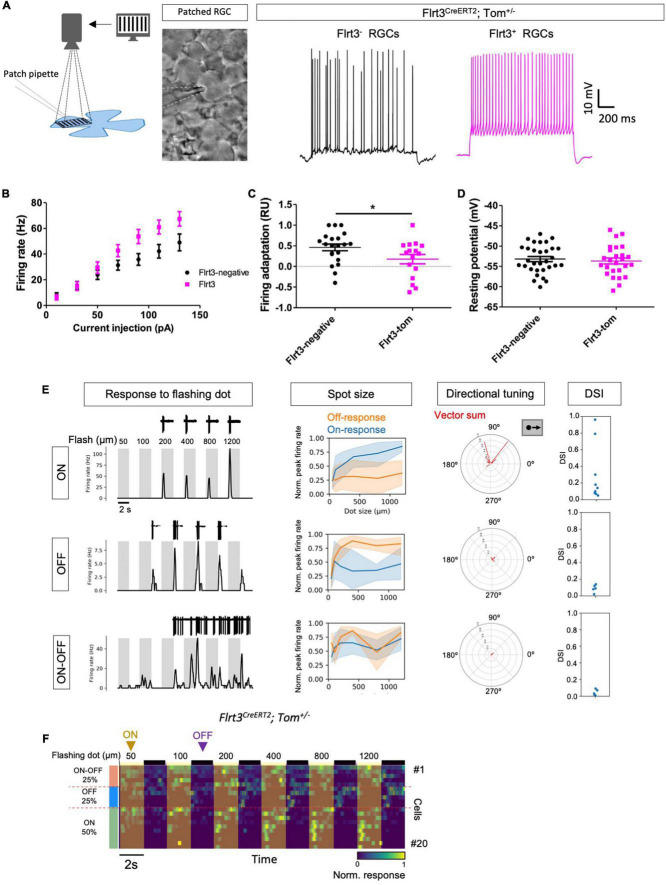
Flrt3-Tom-RGCs are mainly ON neurons. **(A)** The left panel shows a schematic of the experiment in which an RGC is patch-clamped while different movie patterns are shown with a projector. The right panel shows representative whole-cell current-clamp recordings displaying firing after a step of current injection in Flrt3^+^ and Flrt3^–^ RGCs. **(B)** Plot showing the firing rate (Hz) after increasing current steps. **(C)** Firing adaptation from the recordings in **(A)**. A value for firing adaptation closer to 1 represents cells with a higher adapting firing, while a value closer to 0 is more regular (see section “Materials and Methods”). *N* = 16–21 cells from 2 retinas and 2 animals. Two-tailed unpaired *t*-test. *P < 0.05. **(D)** Resting potential (mV) in Flrt3^+^ and Flrt3^–^ RGCs. **(E)** The first column of data shows representative responses (ON, OFF, and ON-OFF) in cell-attached mode to a flashing dot of increasing sizes with ON (gray) and OFF (white) over the center of each cell; the top of the plots show representative recordings in cell-attached mode. The second column shows the firing rate after the flashing spot stimulus. The third and fourth columns show the directional tuning and direction selectivity index (DSI), respectively, after the moving dot stimulus. The results show the responses to dots moving across the center of the RGCs in different directions. Polar plots represent the number of spikes fired for bars moving in each of the eight directions. **(F)** Heatmap plot showing the firing rate of 20 Flrt3-Tom-RGCs during the flashing dot stimulus. The yellow and black lines on top represent the dot flashing ON and OFF, respectively. Flrt3-Tom-RGCs are 50% ON, 25% OFF, and 25% ON-OFF. *N* = 20 cells from 6 animals. Neurons of unknown identity were discarded (*N* = 4 cells, data not shown).

**FIGURE 4:**
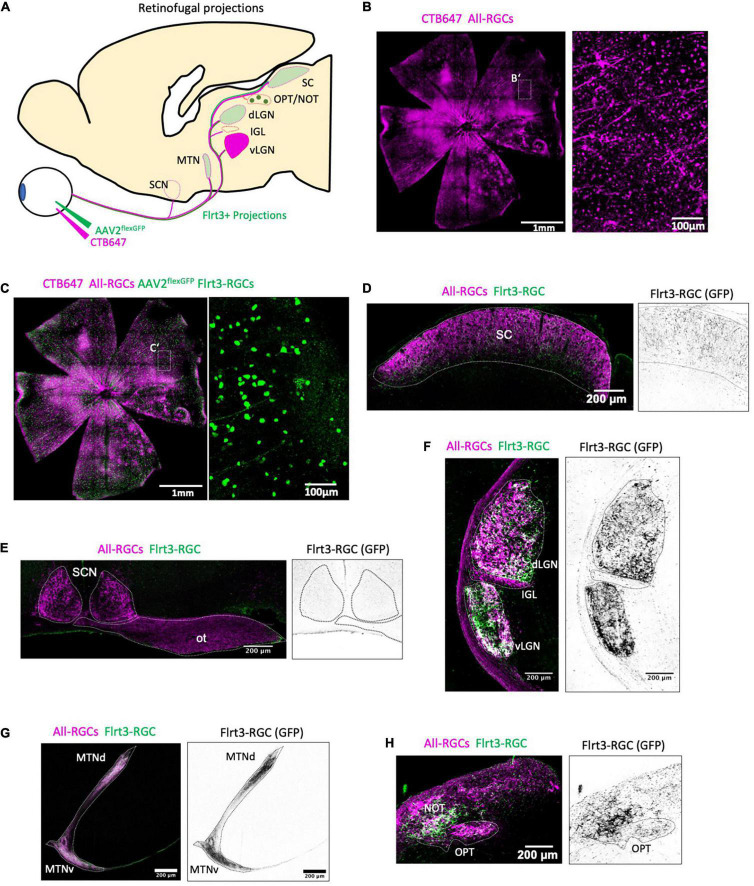
Flrt3-RGCs projections in the mouse brain. **(A)** Scheme showing intravitreal injection of CTB647 and the cre-dependent AAV2-flex-GFP virus in Flrt3-CreERT2 mice. Representative images from one animal. **(B,C)** Representative whole mount retinas showing CTB647 labeled RGCs (magenta) and GFP expressing RGCs (green). **(B)** Shows an overlay of CTB647 and GFP. The right panel shows a zoom from each of **(B’,C’)**, respectively. **(D–H)** Specific projections of Flrt3 RGCs (green) and all RGC (magenta) axons in the **(D)** superior colliculus (SC), **(E)** SCN and optic tract (ot), **(F)** dLGN, IGL and vLGN, **(G)** MTN, **(H)** NOT and OPT.

**FIGURE 5:**
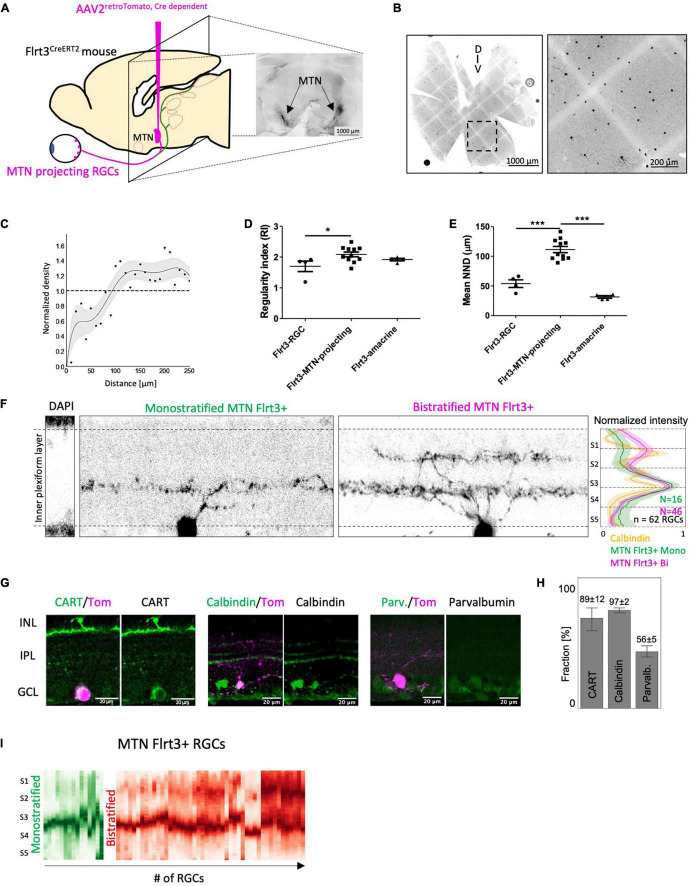
MTN-projecting Flrt3-RGCs are a homogeneous downward ON-DSGC subpopulation. **(A)** Stereotaxic injection of AAV2^retro–cre–Tomato^ in the MTN of Flrt3-CreERT2 mice to identify Flrt3-RGCs projecting to the MTN. The image on the right shows a representative MTN-injection site after 3 weeks of viral expression. *N* = 4 mice. **(B)** Whole mount retina showing the distribution of MTN-projecting Flrt3-RGCs. **(C)** Normalized density profile of MTN-projecting Flrt3-RGCs. *N* = 50 ROIs from 11 retinas and 8 animals. **(D)** Regularity index plot for Flrt3-RGC, Flrt3-MTN-projecting-RGC and Flrt3-amacrine cells. *N* = 4–11 ROI from different retinas of 3–4 mice (as shown in **(B)**. One-way ANOVA Tukey’s Multiple Comparison Test, *P < 0.05. **(E)** Average nearest neighbor distance (μm), calculated from same ROIs as in **(D)**. One-way ANOVA Tukey’s Multiple Comparison Test, ***P < 0.001. **(F)** Orthogonal section through the IPL to visualize the stratification pattern of MTN-projecting Flrt3-RGCs. The right panel shows the average normalized stratification pattern of Calbindin (orange), mono- (green, *n* = 16 cells from 3 animals, 26%) and bistratified MTN-projecting Flrt3-RGCs (magenta, *n* = 46 cells from 3 animals, 74%). **(G)** Immunostaining for CART, Calbindin and Parvalbumin in a retinal sections of MTN injected mice from **(A)**. Tomato signal (magenta) shows MTN-projecting Flrt3-RGCs. **(H)** Fraction (%) of MTN-projecting Flrt3-RGCs positive for CART, Calbindin and Parvalbumin. *N* = 60–66 ROIs for each marker from 3 retinas. **(I)** Normalized stratification patterns of mono- (green) and bistratified (red) MTN-Flrt3-RGCs in the inner plexiform layer, from **(F)**. *N* = 46 cells from 3 animals.

## Results

### Flrt3 Is Expressed in a Subpopulation of Retinal Ganglion Cells

In previous work, we observed Flrt3 expression in the early postnatal retina (Seiradake et al., 2014). Additionally, all three members of the Flrt family of cell-adhesion molecules (Flrt1, 2, and 3) were found to be expressed in the adult retina (Visser et al., 2015). Using our Flrt1^LacZ^, Flrt2^lacZ^, and Flrt3^lacZ^ reporter mouse lines, we confirmed that all three Flrts are expressed in a subpopulation of neurons in the ganglion cell layer (GCL) (Supplementary Figures 1A–F and data not shown). Since Flrt3 showed a sparser expression pattern in the GCL than Flrt1 and 2, we decided to focus on characterizing the Flrt3 population (Supplementary Figures 1A,B). To gain genetic access to the Flrt3-RGCs, we generated a tamoxifen-inducible Flrt3-CreERT2 knock-in line (Figure 1A). By crossing the Flrt3-CreERT2 line to a tdTomato reporter line, we confirmed that Flrt3-Cre expression matched Flrt3^LacZ^ expression in the brain (Supplementary Figures 2A–C). Immunostainings using the pan RGC marker RBPMS [RNA-binding protein (Rodriguez et al., 2014)] showed that 22.7% of all Flrt3+ neurons in the GCL were RGCs, while 77.3% were amacrine cells (Figures 1B,C). Conversely, Flrt3-RGCs represented a sparse population of about 4.6% of all RGCs. Retinal sections (vibratome cut sections orthogonal to the retinal cell layers) of the Flrt3-CreERT2-Tomato line showed that Flrt3 was not only expressed in the GCL but also in the inner nuclear layer (INL) (Figure 1D and Supplementary Figure 1F). Flrt3+ amacrine cells, RGCs, and possibly also bipolar cells of the INL and GCL contribute to the stratification pattern observed within the IPL. The peak stratification is located between the two ON and OFF ChAT bands (Voigt, 1986) (Figure 1D). Moreover, the Flrt3-CreERT2 line also labels a population of horizontal cells in the OPL (data not shown).

A relatively homogeneous distribution and small fraction of Flrt3-RGCs suggested that only a limited number of functionally specific RGCs types were labeled (Figures 1E,F). To examine if Flrt3-RGCs represented an anatomically homogeneous subpopulation with a regular spacing, we analyzed their spatial arrangement based on density recovery profiles (DRP) (Supplementary Figure 2D) (Rodieck, 1991). We found that Flrt3-RGCs were distributed across the whole retina, with a peak density in the ventral regions (Figure 1E). However, DRP analysis showed random distribution in both Flrt3-RGCs (Figure 1F) and Flrt3-amacrine cells (Supplementary Figure 2E). In summary, these results suggest that Flrt3 labels more than one functional RGC and amacrine subpopulation.

### Morphological Characterization Indicates at Least Four Flrt3-RGC Subpopulations

The depth of dendritic stratification in the IPL is a good feature to classify functional RGC subpopulations (Seung and Sümbül, 2014). Thus, we used the Flrt3-CreERT2-Tomato line to label the morphology of Flrt3^+^ RGCs and amacrine cells by electrophoretic injection of fluorescent dyes in random locations of the retina (Lucifer yellow and Alexa 488, Figures 2A,D,E). We divided the IPL into five layers (S1–S5) and used this to assign the dendritic distribution of the individual labeled cells (Figure 2B). We found that Flrt3-RGCs consist of 4 subtypes: 53% of all Flrt3-RGCs were bistratified in the ON and OFF layers S2 and S4, 37% stratified in the ON layers (2 subtypes) S4 or S5, and a small fraction (∼10%) stratified only in the OFF layer S1 (Figure 2C).

To further characterize the Flrt3-RGC subpopulations, we performed immunostainings against known RGC markers. The strongest co-labeling of Flrt3+ RGCs (RBPMS+) was found with Calbindin (28 ± 2.7%), followed by Parvalbumin (19 ± 3.6%), and then the ON-OFF direction selective ganglion cell marker CART (22 ± 4.8) (Supplementary Figures 3A–F, mean ± SEM). Overall, none of the markers labeled all Flrt3+ RGCs, suggesting that Flrt3 labels a new combination of RGC subtypes. Importantly, it has been shown in mice that parvalbumin and calbindin label at least eight and ten (PV1–PV8), respectively, morphologically different RGC types differing in their stratification pattern and dendritic field size (Kim and Jeon, 2006; Gu et al., 2016). CART positive RGCs are known to label the four ON-OFF direction selective ganglion cells (ooDSGC) that can be distinguished using combinatorial staining of Cadherin 6, MMP17, and Collagen 25a1 (Kay et al., 2011).

### Electrophysiological Characterization Revealed Three Major Response Patterns

To physiologically characterize Flrt3-RGCs, we performed patch clamp recordings from Flrt3-Tom mice, showing different movie patterns (e.g., moving bars, stripes, or dots) directly onto the retina to monitor light-evoked firing activities (Figure 3A). In an initial evaluation, we analyzed the firing properties of Flrt3-RGCs by injecting current pulses with different intensities in whole-cell current-clamp mode. We recorded 21 Flrt3-negative and 16 Flrt3-positive RGCs from random retina regions. Interestingly, Flrt3-RGCs showed a higher firing rate than those of Flrt3-negative RGCs (Figures 3A,B). Moreover, when comparing the temporal features of firing patterns, Flrt3-RGCs showed non-adaptive and regular firing patterns (Flrt3-negative 0.46 ± 0.08, Flrt3-Tom 0.18 ± 0.11, *t*-test *p < 0.05) (Figure 3C). The membrane potential of Flrt3 and Flrt3-negative RGCs did not show any significant differences (Flrt3-negative −53.21 ± 0.63, Flrt3-Tom −53.69 ± 0.74) (Figure 3D).

To evaluate the visual responses of Flrt3-RGCs, we performed cell-attached recordings to analyze the light-evoked firing activity in response to a set of different movie patterns: static flashing spot with different sizes (50–1,200 μm), and moving bars (Figures 3E,F). The responses to static flash stimulus revealed that Flrt3-RGCs included ON (50%), OFF (25%), and ON-OFF (25%) cells. A subset of ON cells showed direction selectivity to upward movements (2 out of 10 cells) (Figure 3E). Within our sample size no OFF and ON-OFF cells showed any direction selectivity (Figure 3E).

### Flrt3-RGCs Project to Nuclei in the Accessory Optic System

Retinal ganglion cells project to one of more than 50 different target regions in the mouse brain (Martersteck et al., 2017), and the projection patterns vary depending on RGC subtypes (Lawrence and Studholme, 2014). To analyze the projection pattern of Flrt3-RGCs, we performed intravitreal virus injections using a Cre-dependent GFP expressing AAV to specifically label the projection targets of Flrt3 RGCs, and compared it to the overall projection pattern of all RGCs using CTB647 (Figures 4A–C). The expression pattern showed non-specific innervation of the superior colliculus and LGN (Figures 4D,E). No Flrt3-RGCs axons were found in the intergeniculate leaflet (IGL) or the suprachiasmatic nucleus (SCN) (Figures 4E,F). Interestingly, Flrt3 RGCs projected to the medial terminal nucleus (MTN) and nucleus of the optic tract (NOT) (Figures 4G,H) which are part of the AOS and are known to be essential in driving the optokinetic reflex.

### Medial Terminal Nucleus Projecting FLRT3 Retinal Ganglion Cells Are ON-DSGC

Based on the finding that Flrt3-RGCs project to the MTN of the AOS, we hypothesized that Flrt3-RGCs contain a subpopulation of direction selective ganglion cells (DSGC) encoding one of the four cardinal axes. To specifically label only Flrt3-RGCs projecting to the MTN, we injected a retrograde Cre-dependent AAV-Tomato into the MTN of Flrt3-CreERT2 mice (Figure 5A). After 3 weeks of viral expression, we were able to identify MTN-projecting Flrt3-RGCs in the retina (Figure 5B). Density recovery profile analyzes revealed a regular distribution of MTN projecting Flrt3-RGCs, indicating a homogenous subpopulation (Figure 5C). Indeed, the regularity index (RI) (see section “Materials and Methods”) and mean nearest neighbor distance (NND) showed higher values for MTN-projecting Flrt3-RGCs (RI = 2.08 ± 0.07; NND = 111.4 ± 5.29) than for total Flrt3-RGCs (RI = 1.7 ± 0.17; NND = 54.04 ± 6.44) and Flrt3-amacrine cells (RI = 1.92 ± 0.04; NND = 31.57 ± 2.22) (Figures 5D,E), suggesting that MTN projecting Flrt3-RGCs consist of a single RGC subtype.

The majority of MTN-projecting Flrt3-RGCs bistratified in the S4 ON and S2 OFF layer (∼74%) of the inner plexiform layer (IPL) and overlapped with the two outer bands of Calbindin (Figure 5F). The remaining MTN-projecting Flrt3-RGCs mono-stratified in the S4 layer and overlapped with the inner most band of Calbindin (Figure 5I). Immunostainings showed that most MTN-projecting Flrt3-RGCs are positive for the ON-OFF DSGC marker CART (89 ± 11%, *n* = 69 cells) and Calbindin (97 ± 2%, *n* = 98 cells) (Figures 5G,H), which have been shown to label at least 10 morphologically distinguishable RGC types (Gu et al., 2016). Interestingly, among Calbindin-containing RGCs, Gu et al. (2016) reported that calretinin-containing RGCs include only 1 type (“CB3” type) that is bistratified in the S2/S4 layers of the IPL, similar to what we show with our MTN-projecting Flrt3-RGCs (Gu et al., 2016). Approximately 56 ± 5% (*n* = 49 cells) co-labeled for the calcium-binding protein parvalbumin (Figures 5G,H), which has been shown to label 8 morphologically distinct RGC types (PV1-8), and only the PV3 subtype shows the same bistratification pattern as most of the MTN-projecting Flrt3-RGCs (Yi et al., 2012). These data indicate that the known CB3 and PV3 RGCs are possible candidates for the cell types of MTN-projecting Flrt3-RGCs.

**FIGURE 6:**
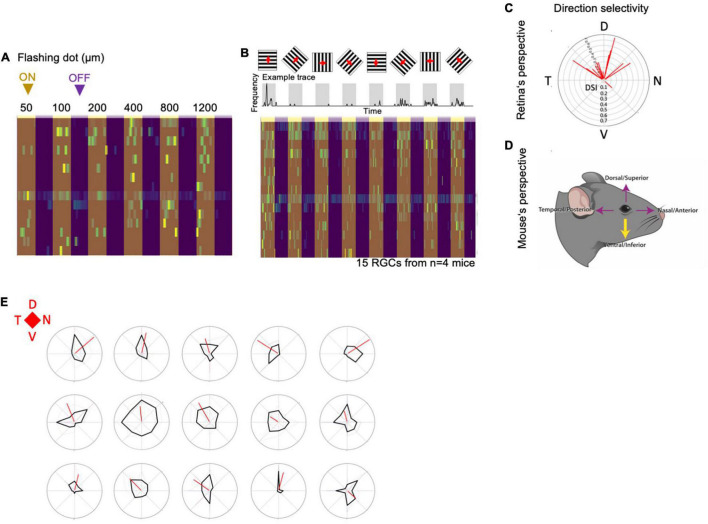
MTN-projecting Flrt3-RGCs are a homogeneous downward ON-DSGC subpopulation. **(A)** Heatmap plot showing the frequency of firing in cell-attached MTN-projecting Flrt3-RGCs while showing a flashing dot with different diameters (50–1,200 μm). **(B)** Top: Scheme representing the recordings of MTN-projecting Flrt3-RGCs firing rate while displaying a moving grid in eight different directions. The frequency plot shows the average frequency of firing. Bottom: The heatmap plot shows the individual frequencies for each neuron patched for each moving grid displayed. *N* = 15 RGCs from 4 animals. **(C)** Polar plot showing the direction selectivity of the RGCs recorded from the retina’s perspective. *N* = 15 RGCs from 4 animals. **(D)** Cartoon showing the mouse’s perspective of the moving object. **(E)** Direction selectivity plots of fifteen different MTN-Flrt3-RGCs. *N* = 15 cells from 4 animals.

Although the majority of MTN-projecting Flrt3-RGCs had bistratified dendrites in the ON and OFF layers, the light-evoked firing activity monitored by cell-attached recordings revealed that MTN-projecting Flrt3-RGCs are physiologically ON cells, responding to luminance increments of static flashes (50–1,200 μm) (Figure 6A). Interestingly, almost all MTN-projecting Flrt3-RGCs (14 out of 15) were direction selective cells, and preferentially responded to an upward-moving grating (Figures 6B,C,E), corresponding to ventrally moving objects in the living animal (Figure 6D). Together, these results indicate that MTN-projecting Flrt3 RGCs represent a homogeneous AOS projecting direction-selective RGC subpopulation.

## Discussion

In this work, we generated a new Flrt3-CreERT2 knock-in mouse line to genetically target and characterize Flrt3 positive RGCs in the mouse retina. We found that Flrt3-RGCs represent a small subpopulation of ∼4.6% of all RGCs. Based on their stratification patterns, Flrt3-RGCs can be subdivided into four morphologically and three functionally distinct subtypes. Electrophysiological recordings showed that Flrt3-RGCs have more non-adaptive and higher firing rates than Frt3-negative RGCs. Fifty percent of Flrt3-RGCs are ON-responding cells when they are stimulated with a static flashing light. Retinofugal projection and electrophysiological analyses revealed that the functional ON subtype of the Flrt3 population projects to the MTN. Histologically, MTN-projecting Flrt3-RGCs bistratify in the IPL and express the ooDSGC marker CART (∼89%) and Calbindin (∼97%). Further analysis showed that MTN-projecting Flrt3-RGCs are direction selective cells, preferring downward direction.

Prior research on MTN-projecting direction-selective RGCs (Dhande et al., 2013) revealed that the Hoxd10-EGFP mouse line includes three ON subtypes, each of which encodes upward, downward or forward motion, and one ON-OFF type encode forward movements. So far, the Fstl4^TM1Mno^ (here referred to as SPIG1) mouse line is the only mouse model that specifically labels the upward direction-selective ON-mono-stratifying MTN-RGCs. For the remaining 3 subtypes, no genetic marker exists (Yonehara et al., 2009; Dhande et al., 2013). In contrast to our MTN-projecting Flrt3-RGCs, downward selective cells in Hoxd10 mice show an ON mono-stratifying pattern in the IPL. Interestingly, Dhande et al. described a small (11%) population of RGCs in the Hoxd10 line that co-localized with the ON-OFF DSGC marker CART. However, the cell type was not further characterized. Another genetic line, Pcdh9-Cre, also labels MTN projecting ON RGCs. Like the MTN projecting Flrt3+ cells, the Pcdh9-Cre line labels 2 subtypes that either stratify mostly in the ON S4 layer, or show weaker stratifications in the S2 OFF layer. Both subtypes, however, respond to upward moving stimuli. In contrast to the MTN projecting Flrt3+ RGCs, the Pcdh9 ON-DS cells show a transient OFF response to the trailing edge of the moving stimulus (Lilley et al., 2019). Overall, this data suggests that Flrt3-RGCs could represent the CART positive RGCs of the Hoxd10 mouse line and thus describe a new and as yet uncharacterized MTN-projecting RGC population.

The co-stratification of MTN projecting Flrt3-RGCs within the same layer of the ON and OFF starburst amacrine (SAC) dendrites suggests that their direction selectivity toward downward moving objects is mediated by modulating GABAergic and cholinergic input from SACs (Mauss et al., 2017). However, we found a discrepancy between the stratification pattern and physiological response properties. Although MTN-Flrt3-RGCs showed a bi-stratifying ON-OFF morphology within the IPL, they lacked any functional OFF response. The small OFF responses seen despite the ON-OFF dendritic bistratification could be caused by the offset of OFF excitatory inputs by inhibitory inputs or presynaptic inhibition of OFF bipolar cells (Jacoby, 2015; Nath and Schwartz, 2016). Indeed, the same discrepancy between dendritic bi-stratification and ON dominant responses has been revealed in orientation selective RGCs (Nath and Schwartz, 2016), suppressed-by-contrast RGCs (Jacoby, 2015) and MTN/SC projecting ON DSGCs (Gauvain and Murphy, 2015), in which OFF dendrites did not receive any excitatory inputs. The absent OFF response could also be explained by the shorter dendritic ramification in the OFF layer in comparison with the longer projection into the ON layer (Figure 5F; Nath and Schwartz, 2016).

## Conclusion

Based on the light responses of RGCs and basic anatomical criteria, we found a new genetic marker to identify a novel FLRT3^+^ cell-type in the mouse retina, corresponding to an ON downward-selective MTN-projecting RGC. Like the Hoxd10 mouse, which identified an additional ON-OFF RGC that projects to the NOT of the AOS, our findings add a homogeneously distributed ON RGC-type that projects to the MTN of the AOS. Interestingly, this MTN-projecting FLRT3^+^ cell-type is positive for CART, a marker for ON-OFF RGCs. Moreover, this RGC-type bistratified in the ON and OFF layer, but the electrophysiological recordings showed that they fire only when the light is ON, probably because the stratification pattern is larger in the ON layer compared to the OFF layer of the IPL.

In conclusion, the current work shows that almost all MTN-projecting Flrt3 cells are: (1) bistratified, (2) ON-dominant response, and (3) direction selective RGCs. Finally, using intersectional genetics, our Flrt3 line in combination with the Parvalbumin, Calbindin or CART transgenic mouse lines could provide functional access to these RGCs, which would enable us to study the function of the downward selective Flrt3-RGCs *in vivo.*

## Author’s Note

The retinal ganglion cells (RGCs) in the retina are the only cells that transmit visual information into the brain. Until now, more than 40 different RGC types have been identified which respond in a very specific way to the same visual stimuli. However, only a small fraction of these can be studied using specific genetic markers. We found that Flrt3 labels a new RGC subpopulation that projects to the medial terminal nucleus (MTN) which is essential to drive reflexive eye movements for retinal image stabilization. Flrt3 labels MTN projecting RGCs that preferentially respond to downward moving objects by increasing their firing rate (ON-response). Thus, Flrt3 provides the first genetic tool for studying downward motion selective RGCs projecting to MTN.

## Data Availability Statement

The original contributions presented in the study are included in the article/Supplementary Material, further inquiries can be directed to the corresponding author/s.

## Ethics Statement

The animal study was reviewed and approved by the Regierung von Oberbayern under the license 55.2-2532.Vet_02-20-10.

## Author Contributions

RK, DT, and TR initiated and conceived the project. CP performed electrophysiological recordings and intracranial injections. AM helped with electrophysiological recordings supervised by KY. TR performed all remaining experiments. PM and LG performed ES cell gene targeting. SI wrote the python analysis script to straighten the curved retina sections for stratification analysis. TR and CP wrote the manuscript with support from all other authors. LG checked the English grammar of the manuscript. All authors contributed to the article and approved the submitted version.

## Conflict of Interest

The authors declare that the research was conducted in the absence of any commercial or financial relationships that could be construed as a potential conflict of interest.

## Publisher’s Note

All claims expressed in this article are solely those of the authors and do not necessarily represent those of their affiliated organizations, or those of the publisher, the editors and the reviewers. Any product that may be evaluated in this article, or claim that may be made by its manufacturer, is not guaranteed or endorsed by the publisher.

## Footnotes

1
https://neurom.readthedocs.io/en/stable/

## Supplementary Material

Supplementary Figure 1 Flrt2 and 3^LacZ^ lines express Flrts in a subpopulation of neurons in the ganglion cell layer. **(A)** Representative retina from Flrt3-LacZ animals. The immunostaining in the right panel shows Flrt3 cells (light blue, Xgal) and RGCs (green, RBPMS). White asterisks indicate Flrt3+ RGCs. Scale bar = 25 μm. **(B)** Top: percentage of Flrt3^+^ RGCs and amacrine cells in the ganglion cell layer. Bottom: percentage of Flrt3^+^ cells among RGCs. Scale bar = 25 μm. **(C,D)** Same figures as **(A,B)**, but with Flrt2-LacZ mice. White asterisks indicate Flrt2+ RGCs. **(E,F)** Retina sections from Flrt2-LacZ and Flrt3-LacZ mice, respectively, with Xgal staining at different developmental time points.

Supplementary Figure 2 Flrt3-CreERT2 expression matches Flrt3^LacZ^ expression in the brain. **(A)** Coronal brain slices of Flrt3-CreERT2*;* Tom^±^ mice, from anterior to posterior. **(B)** Zoomed in view of different regions from **(A)** (dashed boxes). **(C)** Comparison between Flrt3-Tom and Flrt3/LacZ mice. **(D)** Algorithm showing how the DRP values were calculated. Rectangular regions of the retina were selected to create an autocorrelogram. Annuli around the center of the autocorrelogram were drawn to calculate the binned density from the center to the periphery and represented as the density recovery profile. **(E)** Random distribution of Flrt3-amacrine cells. Left panel: Flrt3-amacrine cell distribution in a ROI. Each dot represents a Flrt3+ amacrine cell. Right panel: Density recovery profile of Flrt3+ amacrine cells showing a random distribution. Graph shows mean value ± SEM. Line represents a 7th order fit of the mean values. Shaded area is the 99% confidence interval. *N* = 14 ROIs from 4 retinas and 2 mice.

Supplementary Figure 3 Immunostainings showing some overlap with other known RGC markers. **(A–F)** Staining for various known markers: Safb1, SMI32, Calbindin, Bm3c, Parvalbumin, and CART (blue) in Flrt3-RGCs (Tom, magenta) and RBPMS (green). The blue arrowhead shows an example of RGC that co-localizes with Flrt3 and the different markers. **(G)** Quantification of the percentage of Flrt3-RGCs expressing the markers shown in **(A–F)**. Mean value ± SEM.
