# Supplementary figures and images for: FLRT3 Marks Direction-Selective Retinal Ganglion Cells That Project to the Medial Terminal Nucleus
Source: Front Mol Neurosci. 2021 Dec 9;14:790466. doi: 10.3389/fnmol.2021.790466 (PMC8696037; doi:10.3389/fnmol.2021.790466)

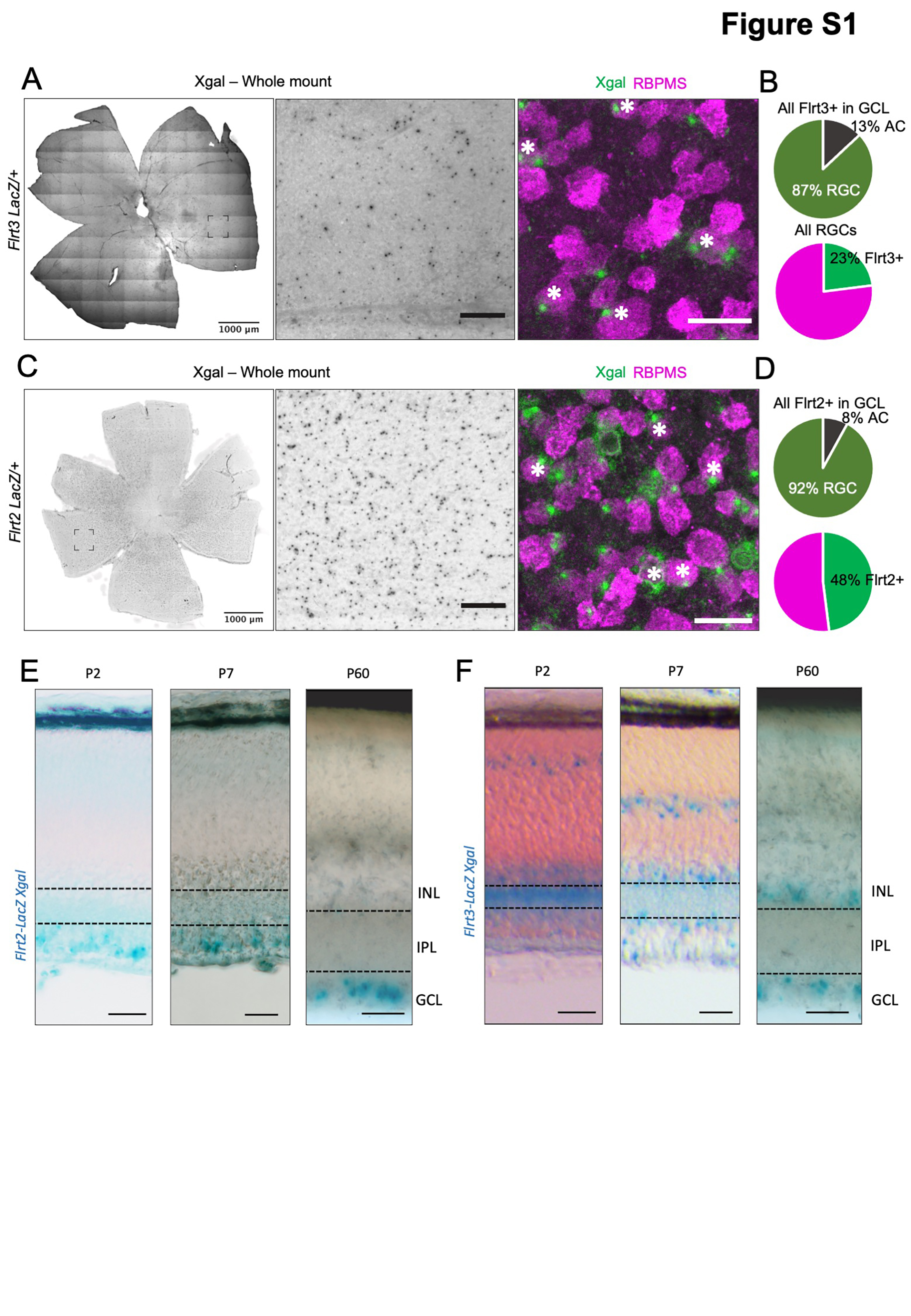

Supplement: Supplementary Figure 1 — Flrt2 and 3LacZ lines express Flrts in a subpopulation of neurons in the ganglion cell layer. (A) Representative retina from Flrt3-LacZ animals. The immunostaining in the right panel shows Flrt3 cells (light blue, Xgal) and RGCs (green, RBPMS). White asterisks indicate Flrt3+ RGCs. Scale bar = 25 μm. (B) Top: percentage of Flrt3+ RGCs and amacrine cells in the ganglion cell layer. Bottom: percentage of Flrt3+ cells among RGCs. Scale bar = 25 μm. (C,D) Same figures as (A,B), but with Flrt2-LacZ mice. White asterisks indicate Flrt2+ RGCs. (E,F) Retina sections from Flrt2-LacZ and Flrt3-LacZ mice, respectively, with Xgal staining at different developmental time points. [file Image_1.jpeg]

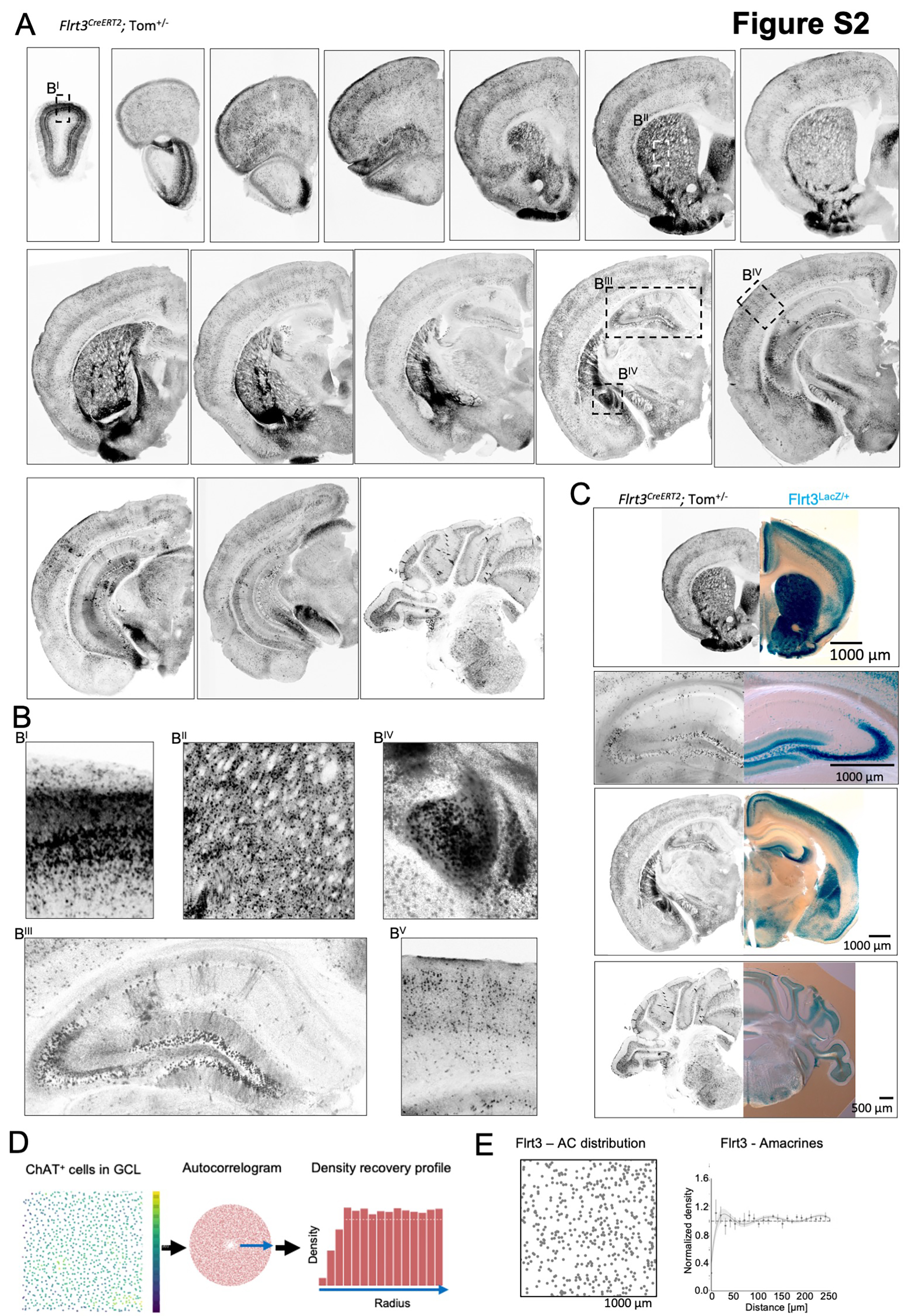

Supplement: Supplementary Figure 2 — Flrt3-CreERT2 expression matches Flrt3LacZ expression in the brain. (A) Coronal brain slices of Flrt3-CreERT2; Tom± mice, from anterior to posterior. (B) Zoomed in view of different regions from (A) (dashed boxes). (C) Comparison between Flrt3-Tom and Flrt3/LacZ mice. (D) Algorithm showing how the DRP values were calculated. Rectangular regions of the retina were selected to create an autocorrelogram. Annuli around the center of the autocorrelogram were drawn to calculate the binned density from the center to the periphery and represented as the density recovery profile. (E) Random distribution of Flrt3-amacrine cells. Left panel: Flrt3-amacrine cell distribution in a ROI. Each dot represents a Flrt3+ amacrine cell. Right panel: Density recovery profile of Flrt3+ amacrine cells showing a random distribution. Graph shows mean value ± SEM. Line represents a 7th order fit of the mean values. Shaded area is the 99% confidence interval. N = 14 ROIs from 4 retinas and 2 mice. [file Image_2.jpeg]

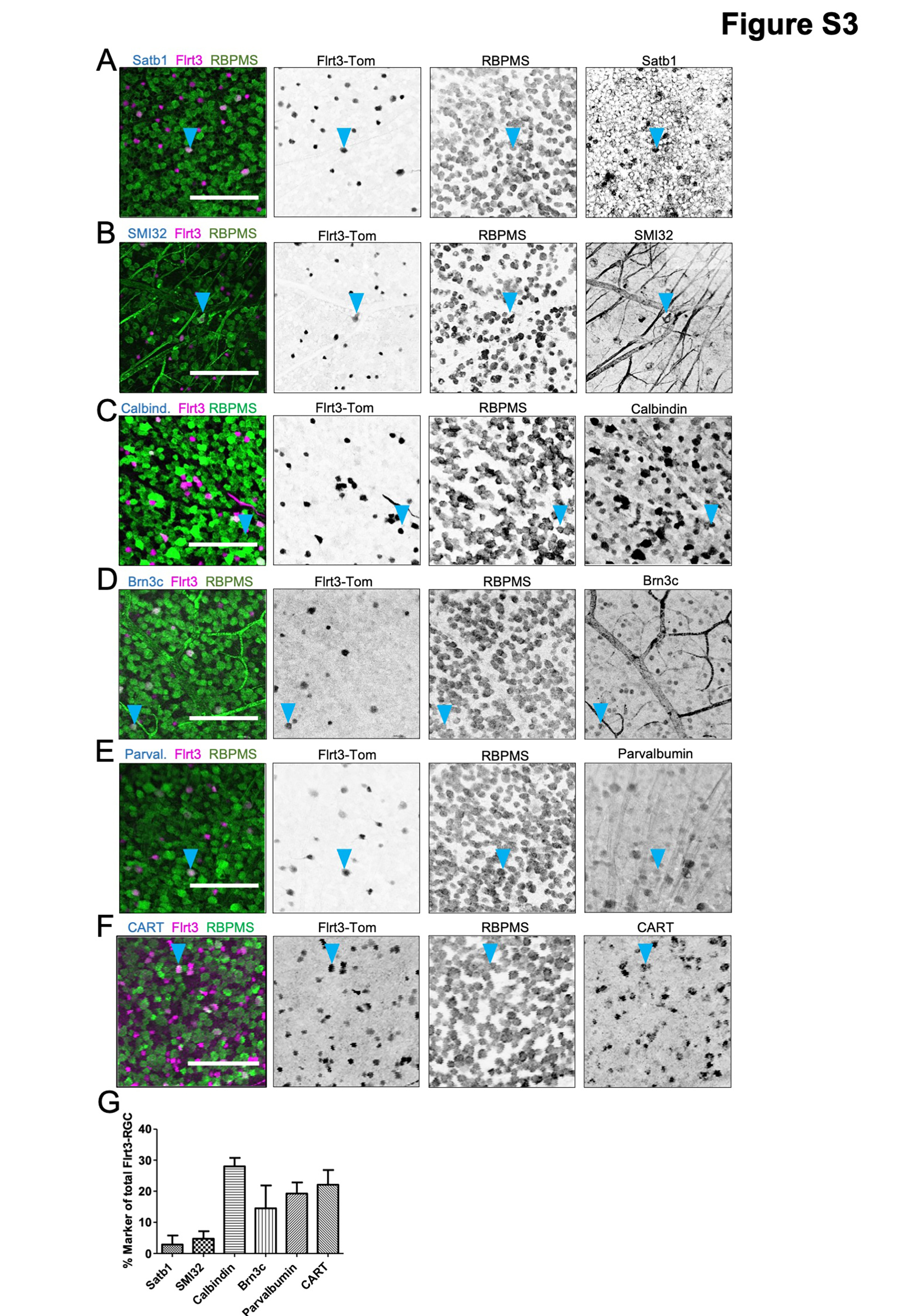

Supplement: Supplementary Figure 3 — Immunostainings showing some overlap with other known RGC markers. (A–F) Staining for various known markers: Safb1, SMI32, Calbindin, Bm3c, Parvalbumin, and CART (blue) in Flrt3-RGCs (Tom, magenta) and RBPMS (green). The blue arrowhead shows an example of RGC that co-localizes with Flrt3 and the different markers. (G) Quantification of the percentage of Flrt3-RGCs expressing the markers shown in (A–F). Mean value ± SEM. [file Image_3.jpeg]
